# Gut check: antimicrobial stewardship opportunities in intra-abdominal infections

**DOI:** 10.1017/ash.2025.10169

**Published:** 2026-02-02

**Authors:** Ritika Prasad, Radhika Arya, Natalie Medvedeva, David Ha, Marisa Holubar

**Affiliations:** 1 Division of Infectious Diseases and Geographic Medicine, Stanford University School of Medicine, Stanford, CA, USA; 2 Stanford Antimicrobial Safety and Sustainability Program, Stanford Health Care, Stanford, CA, USA

## Abstract

Intra-abdominal infections (IAIs) are common in both the inpatient and outpatient setting but are not often a target for antimicrobial stewardship programs (ASP). However, IAIs provide ASPs an opportunity to translate evidence into practice while also addressing empiric broad-spectrum antibiotic use and establishing relationships with surgical stakeholders. In this review, we analyze five areas of emerging evidence within this heterogeneous field that merit close attention from ASPs, including spontaneous bacterial peritonitis prophylaxis, antibiotic management of appendicitis and biliary tract infections, and optimal amoxicillin-clavulanate and metronidazole dosing.

## Introduction

As antimicrobial stewardship matured as a field, programs shifted from drug-focused to syndrome-based approaches to better support and communicate with primary teams. Respiratory and urinary tract infections are commonly targeted while other more heterogenous categories, like intra-abdominal infections (IAIs), are not.

However, IAIs fit the characteristics of an antimicrobial stewardship program (ASP) priority area—they are common, frequently managed with empiric broad-spectrum antibiotics, and benefit from multi-disciplinary care. In addition, the evidence base that informs the antibiotic management of IAIs is evolving. For example, two pivotal trials, STOP-IT and Dura-POP, support shorter durations of therapy after source control is achieved.^1,2^ One institution published their subsequent experience and demonstrated incomplete practice change, suggesting more work was needed.^3^

ASPs excel at translating evidence into practice, and it may be time to prioritize stewardship interventions for IAIs. This review highlights five areas within this heterogeneous field that merit close attention from ASPs.

## Spontaneous bacterial peritonitis (SBP) prophylaxis

### Primary and secondary SBP prophylaxis

AASLD Guidelines recommend lifelong secondary spontaneous bacterial peritonitis (SBP) prophylaxis for all patients with prior SBP and primary prophylaxis for high-risk patients (ie, patients with advanced cirrhosis and low ascites protein (<1.5 g/dL) with either impaired renal function or hyponatremia).^4^

In recent years, this approach to SBP prophylaxis has been questioned. A 2020 Cochrane meta-analysis of 23 RCTs found no significant benefit of primary or secondary SBP prophylaxis on mortality or number of serious adverse events, however noted the underlying poor quality of included trials.^5^ Concurrently, concerns regarding toxicity and antibiotic resistance have intensified. One retrospective Veterans Affairs (VA) study (*n* = 7,553) found that among patients who were admitted for a first SBP case, those who received primary prophylaxis had a higher proportion of drug-resistant organisms compared to those who received no primary prophylaxis.^6^ Another retrospective VA study (*n* = 11,381) found that patients who received secondary SBP prophylaxis had higher rates of SBP recurrence in multivariate analysis compared to those who received no prophylaxis. Of the 100 patients with available microbiologic data, those who received fluoroquinolone secondary prophylaxis had higher rates of fluoroquinolone-resistant isolates compared to those who received no prophylaxis.^7^

Given this data, clinicians are re-evaluating both primary and secondary prophylaxis. The ongoing ASEPTIC trial may provide clarity.^8^ In the meantime, Markley et al. propose revising institutional SBP prophylaxis guidelines to reflect the limited evidence of benefit and growing evidence of harm.^9^

### SBP prophylaxis in patients with cirrhosis and upper gastrointestinal bleed

In patients with cirrhosis and upper gastrointestinal bleeding (UGIB), antibiotic prophylaxis is the standard of care in Western countries. This practice is based on a meta-analysis of five studies of patients with cirrhosis and gastrointestinal bleeding (*n* = 534), in which short antibiotic durations (mostly oral norfloxacin) were associated with reduced rates of infections, bacteremia and/or SBP, and improved survival.^10^ The AASLD 2021 guidelines recommend intravenous ceftriaxone due to increasing fluoroquinolone resistance.^4^

Recent studies question this practice. A randomized controlled trial from India included 180 hemodynamically stable patients with Child-Pugh A cirrhosis admitted with UGIB and found no difference in infections at 5 days or 6-week mortality between those who received ceftriaxone or placebo.^11^ One retrospective observational study (*n* = 790) in Japan included patients with esophageal variceal bleeding who received emergency endoscopic variceal ligation and found no differences in the primary composite outcome of 6-week mortality, 4-week rebleeding or 4-week SBP onset in those who received antibiotics compared to those who did not.^12^ A smaller Japanese retrospective cohort study (*n* = 150) of patients admitted with acute variceal bleed also found no differences in bacterial infection, in-hospital mortality, 5-day rebleeding rate, and 30-day emergency re-admission in those who received antibiotics compared to those who did not.^13^ These findings may reflect improved modern endoscopic hemostasis techniques and UGIB survival and suggest that further work is needed to evaluate necessity of SBP prophylaxis in UGIB.

While awaiting more data, ASPs can also target SBP prophylaxis duration in patients with cirrhosis and UGIB. National guidelines recommend antibiotics only until bleeding resolves and the patient stabilizes, up to a *maximum* of 7 days, not a fixed 7-day duration.^4^

## Nonoperative antibiotic management of appendicitis

Surgical practices are increasingly incorporating nonoperative management strategies for acute uncomplicated appendicitis. One non-inferiority randomized trial (*n* = 776) showed that a 10-day course of antibiotics was non-inferior to appendectomy for adult patients with imaging-confirmed appendicitis, including those with perforation and appendicolith, for their primary outcome of 30-day health status as measured by the European Quality of Life-5 Dimensions questionnaire. Most patients (71%) in the antibiotic arm avoided appendectomy and many (47%) avoided hospitalization. Antibiotic choice was determined by the ordering physician with at least 24-hours of intravenous antibiotics followed by oral antibiotics.^14^

The APPAC trial, another multicenter, non-inferiority randomized trial performed in Finland compared a 10-day course of antibiotics to appendectomy in adult patients with uncomplicated appendicitis. Patients in the antibiotic arm received intravenous ertapenem for 3 days followed by 7 days of oral levofloxacin and metronidazole. While the study did not demonstrate non-inferiority of antibiotics for the primary outcome of treatment success at one year, 73% of patients in the antibiotic group avoided appendectomy.^15^ The authors acknowledge that ertapenem was chosen given its recommended use as monotherapy for serious IAIs.^16^ A one-size-fits-all antibiotic choice and duration is, however, likely inadequate, and further studies are needed to evaluate optimal choice and duration of antibiotic therapy.

While studies are ongoing to better individualize the management decisions for patients with appendicitis, including the potential for non-antibiotic conservative management approaches,^17^ current evidence supports a role for antibiotic therapy without surgery in select patients. ASPs should be actively involved in guiding antibiotic selection and duration by incorporating local antibiogram data, side effect profiles, and clinical severity into discussions with surgical colleagues as these treatment strategies continue to evolve.

## Enterococcal and anaerobic coverage in biliary tract infections

### Enterococcal coverage

IAIs, including biliary tract infections, are commonly polymicrobial, but the significance of *Enterococcus* spp. in cultures or the need for empiric treatment is uncertain. Observational studies have not demonstrated a consistent benefit of empiric anti-enterococcal therapy in IAIs, especially those of community onset.^18^ Furthermore, a meta-analysis found no improvement in treatment success or mortality when anti-enterococcal regimens were used for IAIs.^19^ In biliary infections specifically, although *Enterococcus* spp. are frequently isolated in bile cultures (13%–30%),^20^ their pathogenic significance in mixed infections without bacteremia is unclear.

Despite this, broad-spectrum regimens that include enterococcal coverage are commonly used in biliary infections, often driven by national guidelines. The Tokyo Guidelines recommend anti-enterococcal therapy for patients with grade III community-acquired (ie, severe infections associated with organ dysfunction) or healthcare-associated acute cholangitis and cholecystitis.^20^ Similarly, the IDSA guidelines suggest enterococcal coverage in patients with healthcare-associated IAIs, particularly in those with postoperative infections, prior exposure to cephalosporins, immunocompromising conditions, valvular heart disease or prosthetic intravascular devices.^16^

However, traditional risk factors may inadequately predict biliary infections with enterococcal bacteremia. Mussa et al. examined 850 cases of bacteremia due to biliary infections from an existing Spanish cohort; 73 (8.5%) were due to *Enterococcus* spp.^21^ They developed a predictive risk score for enterococcal bacteremic biliary tract infections incorporating five variables: cholangiocarcinoma, biliary prosthesis, hospital-acquired infection, prior carbapenem use, and chronic kidney disease. This score demonstrated a negative predictive value (NPV) of 95% at a threshold of six or more points and outperformed the Tokyo Guidelines risk factors (NPV of 92.5%.) However, both demonstrated low positive predictive values (PPV) (Mussa et al: PPV 20.8%; Tokyo Guidelines: PPV: 11.2%).

While it is still unclear which patients truly benefit from empiric enterococcal coverage, ASPs can play a crucial role in ensuring that broad-spectrum regimens with enterococcal activity (eg, piperacillin-tazobactam) are not routinely used for low-risk patients. More definitive evidence is needed to guide when such coverage is necessary.

### Anaerobic coverage

Anaerobic coverage is often reflexively administered in biliary tract infections but this practice has limited benefit.^22^ In a retrospective analysis of intraoperative biliary cultures, Strohäker et al found that only 10% of 365 patient cultures had anaerobes.^23^ Guidelines recommend anaerobic coverage only in cases of documented anaerobic bacteremia or in patients with biliary-enteric anastomoses.^16,20^

This targeted approach is supported by clinical data. In a randomized controlled trial of 100 patients with cholangitis, Sung et al. compared ciprofloxacin monotherapy to combination therapy of ampicillin, ceftazidime, and metronidazole and found no difference in urgent procedural intervention or mortality.^24^ In a cohort of 87 patients with community-onset biliary tract infections without anaerobic bacteremia, Wu et al. found that definitive therapy without anti-anaerobic coverage was not associated with treatment failure, defined as relapse and 28-day mortality.^25^ In a cohort of patients with biliary tract infections, Simeonova et al. showed no significant difference in 30-day mortality or 90-day relapse between patients who received anaerobic coverage and those who did not.^26^ These findings support current guideline recommendations that routine anaerobic coverage has limited clinical value in biliary infections except in specific situations such as anaerobic bacteremia or presence of biliary anastomoses.

## Amoxicillin/clavulanate dosing

Despite the frequency of oral amoxicillin-clavulanate use in IAI, data supporting dosing is surprisingly elusive. A dose of 875/125 mg orally twice daily or, similarly, 500/125 mg orally three times daily was established as the “standard dosage” based on pivotal trials of lower respiratory and urinary tract infections.^27^ Extrapolating dosing from these trials to IAI may be problematic. For example, respiratory infections, namely community-acquired pneumonia, are largely caused by gram-positive or non-*Enterobacterales* gram-negative organisms (as opposed to *Enterobacterales* in IAI) and urinary tract infection treatment may benefit from the high drug concentration in urine.

### Amoxicillin-clavulanate dosing as step-down therapy for Enterobacterales bacteremia associated with IAI

Few randomized controlled trials for gram-negative bacteremia that reported use of oral beta-lactam agents for step-down therapy disclosed specific drugs and doses.^28–30^ Observational studies signaled an increased risk of recurrence with stepdown to oral beta-lactam agents compared with fluoroquinolones or trimethoprim/sulfamethoxazole.^31,32^ Some hypothesize that inadequate oral beta-lactam dosing may be a contributing factor, though this has not yet been substantiated in clinical study.

The pharmacokinetic/pharmacodynamic (PK/PD) target of beta-lactams in *Enterobacterales* bacteremia is the percentage of time in a dosing interval where free serum drug concentration exceeds the minimum inhibitory concentration (MIC) of the pathogen. Modeling data for the most used doses of 875/125 mg orally twice daily or 500/125 mg orally three times daily suggest adequate exposure for *Enterobacterales* with amoxicillin MICs of < = 1 mg/L. A dose of 875/125 mg orally three times daily may be sufficient for amoxicillin MICs < = 2 mg/L.^33,34^ However, current Clinical Laboratory Standards Institute (CLSI) breakpoints for *Enterobacterales* for amoxicillin-clavulanate define amoxicillin MICs of 8 mg/L or lower as “susceptible.”^35^ While CLSI provides a caveat that these recommendations are solely for the purposes of uncomplicated urinary tract infection treatment, clinicians often erroneously extrapolate these results to other infections, like IAI. Further complicating matters, commercially available susceptibility testing systems may not test MICs as low as 1–2 mg/L. Moreover, this MIC range bisects the wild-type MIC distribution for Enterobacterales, limiting the reliability of such testing.^33^

In addition to 875/125 mg three times daily dosing, various other strategies to escalate dosing to achieve the PK/PD target in the setting of higher MICs have been explored, such as 875/125 mg orally twice daily with additional dose(s) (ie, 1 gram) of oral amoxicillin, and the use of extended-release formulations. However, increasing dosing may not be feasible due to gastrointestinal tolerability as well as saturable amoxicillin absorption—effectively limiting the maximum amount of amoxicillin exposure that can be achieved through oral administration.^36–38^

A final practical issue is the limited availability of direct susceptibility testing, which can lead to the common misconception that ampicillin-sulbactam susceptibility can be used as a proxy because it predicts that of amoxicillin-clavulanate. In fact, ampicillin-sulbactam grossly underestimates amoxicillin-clavulanate susceptibility in *Enterobacterales* given that the two agents are fundamentally different, particularly regarding their beta-lactamase inhibitor.^39^

Taken together, these data suggest that a dosage of 875/125 mg orally three times daily appears to be optimal when amoxicillin-clavulanate is used as step-down therapy for bacteremia due to *Enterobacterales* with MICs < = 2 mg/L. It should be noted that much of the clinical data for *E.* bacteremia are in patients with a urinary infection source so further study in IAI is needed.

### Amoxicillin-clavulanate dosing for non-bacteremic diverticulitis

As opposed to gram-negative bacteremia, the PK/PD correlate for efficacy in non-bacteremic IAI is less well understood. That said, two randomized controlled trials, the DIVER trial and the DINAMO trial, assessed amoxicillin-clavulanate as the sole therapy of uncomplicated diverticulitis. Both trials used amoxicillin-clavulanate dosed at 875/125 mg orally three times daily, though the authors do not explicitly mention why this higher dose was used. The DIVER trial compared outpatient management with 10 days of oral amoxicillin-clavulanate to inpatient management with parenteral antibiotic therapy. They found no difference in their primary end point of readmission and lower healthcare costs in the outpatient group.^40^ The DINAMO trial compared 7 days of amoxicillin-clavulanate to non-antibiotic treatment and found no difference in their primary end point of hospitalization and no difference in secondary end points of emergency department visits and pain control.^41^

Based on the findings of the DINAMO trial and other studies, most patients with uncomplicated diverticulitis generally do not require antibiotics. For cases where amoxicillin-clavulanate is chosen, the DIVER trial supports a dose of 875/125 mg orally three times daily, although it should be acknowledged that twice-daily dosing in this setting has not been studied.

### Amoxicillin-clavulanate dosing in non-bacteremic biliary infections, acute appendicitis, or secondary peritonitis

No randomized trials currently exist for amoxicillin-clavulanate as sole therapy for biliary infections, acute appendicitis, or secondary peritonitis; however, data do exist for stepdown therapy.

In complicated IAI with secondary peritonitis, one randomized controlled trial compared stepdown therapy with oral amoxicillin-clavulanate dosed at 800/114mg twice daily to moxifloxacin after piperacillin-tazobactam initial therapy.^42^ Only 2 of the 315 clinically evaluated patients had bacteremia. Clinical cure rates were similar between the moxifloxacin and beta-lactam group. However, durations of therapy of up to14 days were allowed, and median durations used, including duration of parenteral therapy, were not disclosed. Thus, it is unclear how much oral step-down therapy contributed to efficacy.

In medical management of acute appendicitis, two randomized controlled trials exist for oral amoxicillin-clavulanate step-down therapy. In the COMMA trial, O’Leary et al. used parenteral amoxicillin-clavulanate with step-down to oral amoxicillin-clavulanate dosed at 500/125 mg orally three times daily in both the antibiotic-only and surgical arms.^43^ In the ASAA trial, Ceresoli et al. used ertapenem with step-down down to oral amoxicillin-clavulanate dosed at 875/125 mg orally three times daily.^44^ Of note, while ASAA and several randomized trials that primarily used fluoroquinolone step-down therapy showed non-inferiority of antibiotics-only compared with surgical management, the COMMA trial (which used the lower amoxicillin-clavulanate dose) found inferiority of an antibiotics-only approach in a recent meta-analysis.^45^ These data suggest that 875/125 mg orally twice daily may be acceptable for oral step-down therapy in complicated IAI, with possible preference for 875/125 mg orally three times daily for step-down therapy in non-surgical management of appendicitis.

## Metronidazole dosing

Metronidazole was first approved by the U.S. Food and Drug Administration in 1963 for anaerobic infections (including IAIs), trichomoniasis, and amebiasis. Originally, oral and intravenous dosing of 7.5 mg/kg (approximately 500 mg for a 70-kg adult) every 6 hours was recommended for anaerobic infections.^46,47^ However, it is commonly administered as 500 mg orally or intravenously every 8 hours. Growing evidence, including favorable PK/PD data as well as clinical outcomes data, supports alternative dosing such as 500 mg twice daily.

### Pharmacokinetics and pharmacodynamics

Metronidazole is thought to exhibit concentration-dependent killing.^48^ Its half-life is 8–12 hours and serum concentrations at 12 hours remain above the *in vitro* MIC for most anaerobic pathogens. Given its high peak levels, low protein binding, prolonged half-life, active metabolite, and post-antibiotic effect of greater than 3 hours, there is strong PK/PD justification for 500 mg twice daily dosing.^48,49^

### Alternative dosing in anaerobic infections in adults

Clinical data also support 500 mg twice daily metronidazole dosing. One retrospective study of 200 patients with anaerobic or mixed infections (84% IAI) found no difference in clinical cure rates when either twice or three times daily regimens were used.^50^ Additionally, two retrospective studies focusing on anaerobic bacteremia reported no significant differences in 30-day mortality between the two dosing strategies.^51,52^

Twice daily dosing may also reduce the risk of adverse effects associated with cumulative metronidazole exposure, including peripheral neuropathy, encephalopathy, leukopenia, and pancreatitis.^52,53^

### Alternative dosing for appendicitis in the pediatric population

Although PK/PD data is limited in pediatric patients, a dosing regimen of every 6–8 hours has been historically used.^54^ However multiple centers favor once-daily dosing for appendicitis. In a prospective study of children with appendicitis, metronidazole administered once daily at 30 mg/kg per dose achieved AUC target attainment for *Bacteroides fragilis* with an MIC of 2 mcg/mL or less.^54^ A pediatric prospective randomized trial comparing once daily metronidazole and ceftriaxone to ampicillin, gentamicin, and clindamycin for perforated appendicitis found no differences in abscess rates or wound infections, while noting that the once-daily regimen was more cost-effective.^55^

## Conclusion

Despite the evidence reviewed here, many IAI topics are still hotly debated and in need of quality clinical evidence to support ideal practices that improve patient outcomes and minimize adverse events. These include: *What is the optimal definitive therapy for polymicrobial intra-abdominal abscesses? Do all isolated organisms in culture from an intra-abdominal abscess require antimicrobial coverage? What is the optimal duration of therapy for liver and intra-abdominal abscesses with and without complete source control, and what role does serial imaging play? What is the best way to utilize molecular diagnostic panels for the identification of gastrointestinal pathogens and still avoid treatment of false positive or clinical insignificant results?* There are many more.

These persistent clinical questions also highlight the need for ASP to “lean in” to this complicated field. By doing so, ASPs could demonstrate clinical expertise, strengthen relationships with surgical and interventional colleagues, and broaden their reach within their health systems. Ultimately, engaging in management of IAI offers ASPs a critical opportunity to lead practice-changing stewardship efforts in a complex and evolving area of infectious diseases.
